# Correlations between serum 25-hydroxyvitamin D levels and nailfold microvascular changes in psoriatic arthritis patients with distal interphalangeal arthritis treated with TNF inhibitors

**DOI:** 10.3389/fimmu.2026.1847741

**Published:** 2026-07-06

**Authors:** Daniela Anghel, Claudia Oana Cobilinschi, Oana-Georgiana Prioteasă, Daniela Opriş-Belinski, Ioana Cristina Săulescu, Maria-Laura Groşeanu, Anca Bobircă, Violeta-Claudia Bojincă

**Affiliations:** 1 Department of Internal Medicine 2, Dr. Carol Davila University Central Military Emergency Hospital, Bucharest, Romania; 2 Department of Medico-Surgical and Prophylactic Disciplines, Titu Maiorescu University, Bucharest, Romania; 3 Department of Internal Medicine and Rheumatology, Carol Davila University of Medicine and Pharmacy, Bucharest, Romania; 4 Department of Rheumatology, ‘Sf. Maria’ Clinical Hospital, Bucharest, Romania; 5 Department of Internal Medicine and Rheumatology, ‘Dr. Ion Cantacuzino’ Clinical Hospital, Bucharest, Romania; 6 Department of Internal Medicine, ‘Sf. Maria’ Clinical Hospital, Bucharest, Romania

**Keywords:** 25-OH vitamin D, distal interphalangeal joints, microvascular abnormalities, nailfold videocapillaroscopy, psoriasis, psoriatic arthritis, TNF inhibitor therapy

## Abstract

**Background/objectives:**

The role of microvascular abnormalities in the pathophysiology of Psoriatic Arthritis (PsA) has gained increasing attention. Previous studies have identified significant differences in capillary density and morphology in patients with PsA compared to healthy controls.

**Methods:**

This retrospective observational study included 75 female patients—40 with psoriatic arthritis with distal interphalangeal joint involvement and 35 with biopsy-confirmed cutaneous psoriasis (PsO). Data were extracted from patients’ medical records at two time points—baseline and six months after bDMARDs initiation—with strict inclusion and exclusion criteria applied to reduce selection bias.

**Results:**

Using nailfold videocapillaroscopy (NVC), we observed capillary alterations that correlate with the severity of distal interphalangeal joint involvement. In PsA, improvements in capillary parameters likely reflect the effects of biologic therapy combined with vitamin D supplementation, while in PsO, correcting vitamin D levels appears to be the main factor driving vascular improvement. These findings suggest that microvascular alterations may serve as a potential biomarker for disease activity and severity, particularly in patients with predominant distal interphalangeal involvement. In association with ultrasonography, NVC facilitates early detection of pathophysiological alterations, which could influence symptomatology and disease progression and support more targeted and personalized therapeutic strategies. This integrated approach may allow for timely modifications to therapeutic strategies. Moreover, the study explored the association between 25-hydroxyvitamin D (25-OH vitamin D) status and capillary abnormalities.

**Discussions/conclusions:**

Our results demonstrate that NVC is a valuable non-invasive tool for monitoring microvascular changes in patients with distal interphalangeal-predominant PsA undergoing TNF inhibitor therapy. Moreover, they suggest a potential therapeutic impact of TNF inhibitors on vascular health and support the use of NVC as a complementary method in clinical assessment.

## 1 Introduction

Psoriatic arthritis (PsA) is a heterogeneous inflammatory joint disease associated with psoriasis, which is characterized by frequent involvement of both axial and peripheral joints. The diagnosis is primarily based on the presence of dactylitis, peripheral joint, spinal and entheseal inflammation in patients with a history or current cutaneous or nail psoriasis. A personal history of psoriasis or psoriatic arthritis is also a key diagnostic consideration (1, 2).

Moll and Wright classified psoriatic arthritis into five subtypes based on clinical presentation, ranging from peripheral joint involvement to axial and destructive forms (3). Among its clinical phenotypes, distal interphalangeal (DIP) joint involvement is a distinct and challenging subset, often associated with nail dystrophy and enthesitis. This pattern has been linked to more aggressive disease and functional impairment.

While the overall incidence is comparable between sexes, some clinical subtypes show a sex predilection—distal interphalangeal (DIP) joint involvement, for instance, is more commonly observed in male patients (4, 5).

Moreover, extensive or severe plaque psoriasis is associated with a higher risk of psoriatic arthritis development. This association may reflect a more intense or systemic inflammatory process that extends beyond the skin and involves the joints and entheses (6). The close anatomical and developmental relationship between the nail and the DIP entheses can explain why inflammation in the nail bed may extend to contiguous joint structures (7–9).

Vitamin D has emerged as an important immunomodulatory and vascular regulatory factor. In psoriatic disease, low serum levels have been associated with increased disease activity, impaired immune regulation and heightened systemic inflammation. Moreover, vitamin D deficiency has been linked to endothelial dysfunction and microvascular abnormalities in various autoimmune and inflammatory disorders (10).

In the context of psoriatic arthritis, where both systemic inflammation and microvascular involvement play critical roles in disease pathophysiology, assessing the relationship between vitamin D status and capillary changes provides additional insight into microvascular alterations (11).

Nailfold capillaroscopy is useful in monitoring disease activity and progression, early diagnosis, assessing treatment response and predicting long-term disease evolution. Microvascular changes observed through nailfold capillaroscopy might mirror the inflammation happening inside the synovium, potentially serving as a non-invasive biomarker for disease activity in PsA. Several key findings have been observed in PsA: architectural disorganization (irregular and chaotic distribution), increased tortuosity, and a significant reduction in capillary density compared to patients with psoriasis (PsO). Some features can be detected, such as microhemorrhages, angiogenesis, dilated capillaries or very rarely, isolated megacapillaries, suggesting a “scleroderma-like” pattern (12).

The integration of multiple assessment tools in psoriatic arthritis is essential for achieving a comprehensive understanding of the disease activity. This retrospective observational study investigated the potential role of microvascular assessment as a non-invasive biomarker in psoriatic arthritis (PsA). The study explored the utility of nailfold videocapillaroscopy in identifying microvascular changes in patients with PsA primarily involving the distal interphalangeal joints and assessed whether these vascular alterations were associated with the severity of synovitis.

The primary outcome was the evaluation of the association between videocapillaroscopic abnormalities, including capillary morphology and density and disease activity, as assessed by the Disease Activity index for Psoriatic Arthritis (DAPSA) score and C-reactive protein (CRP) levels.

Secondary outcomes included the assessment of the relationships between videocapillaroscopic parameters, ultrasonographic findings and serum 25-OH Vitamin D levels. Specifically, the study aimed to determine whether lower 25-OH Vitamin D levels were associated with more pronounced alterations in capillary morphology and density and whether these findings correlated with disease activity and entheseal changes. Furthermore, the study evaluated whether the extent of distal interphalangeal joint involvement and entheseal inflammation, as visualized by ultrasound, was associated with systemic inflammatory markers and capillaroscopic abnormalities. We also investigated whether lower baseline vitamin D levels were associated with vascular abnormalities and therapeutic response.

## 2 Materials and methods

### 2.1 Study population

A retrospective observational study was conducted on a cohort of 75 patients, selected based on inclusion and exclusion criteria. Participants were diagnosed either with psoriatic arthritis with distal interphalangeal involvement, according to the CASPAR classification criteria, or with psoriasis confirmed by biopsy.

The patients were enrolled in this study after obtaining written informed consent for data collection during routine clinical assessment in our department (“Carol Davila” Central Emergency Military University Hospital, Bucharest, Department of Internal Medicine 2). The data were collected in chronological order from patients who were hospitalized from January 2022 to February 2025.

The inclusion criteria were as follows - PsA group: patients over 18 years old, diagnosed with psoriatic arthritis with DIP involvement (according to CASPAR), who had clinical and imaging assessments (musculoskeletal ecography and nailfold videocapillaroscopy) at an interval of 6 months before (T1 or baseline) and after TNF inhibitors initiation (T2) as well as serum level testing for 25-OH Vitamin D, and inflammatory markers; PsO group – patients over 18 years old who were diagnosed with psoriasis (through biopsy) and were not receiving treatment with DMARDs (disease modifying anti-rheumatic drugs).

The exclusion criteria were as follows: prior biologic or JAK inhibitor therapy, rheumatic inflammatory diseases other than PsA, chronic illnesses (such as cancers, hepatic and renal failure) and history of significant prior trauma to the fingers (history of finger fractures, surgery involving the distal fingers, crush injuries, severe burns, or repetitive occupational/manual trauma affecting the nailfold area).

The PsA group consisted exclusively of patients who were initiated on TNF inhibitors by their treating physicians and who had not previously been exposed to biologic agents or JAK inhibitors. In contrast, the PsO group included patients who were not receiving treatment with DMARDs (disease-modifying anti-rheumatic drugs). This distinction was intentionally made to minimize the potential confounding effect of prior advanced immunomodulatory therapy on nailfold videocapillaroscopic findings, particularly given previous reports suggesting that early TNF inhibition may alter microvascular abnormalities.

To minimize biases, patients were selected sequentially and clear criteria in selecting patients was used. The study protocol was approved by the local ethics committee (No. CR 884/05 Jan 2025).

The recruited patients received treatment from their physicians according to the national recommendations for the management of PsA or PsO. The TNF inhibitors that were prescribed in PsA included Infliximab, Adalimumab or Etanercept. All patients who had 25-OH Vitamin D deficiency received treatment from their physicians: 4000 IU/day for the first 2 months, followed by 2000 IU/day thereafter.

**Figure 1:**
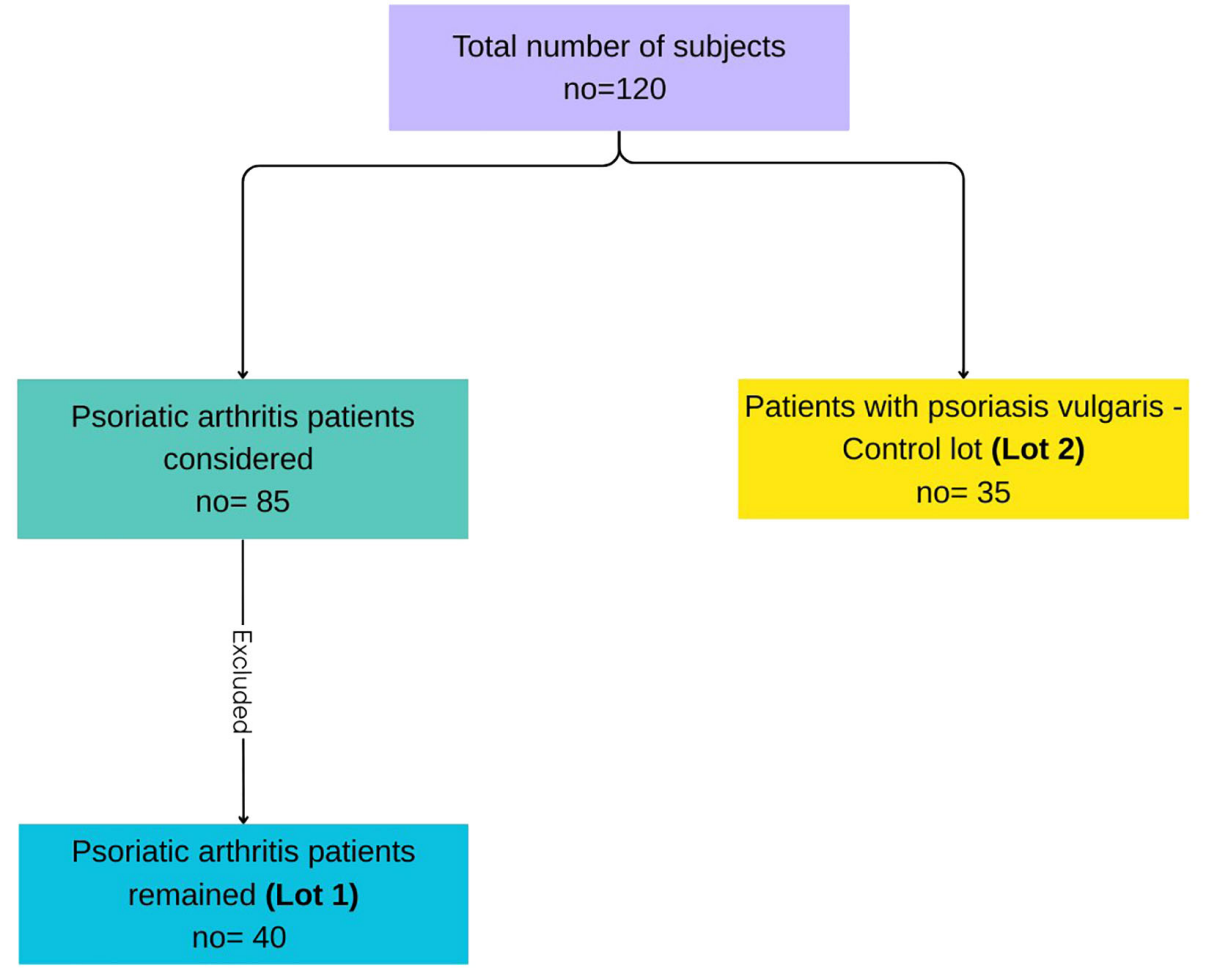
The study population divided into groups.

The cohort was divided into two groups: the psoriatic arthritis group (Group 1/Cohort 1) and the control group (Group/Cohort 2) – **Figure 1**.

### 2.2 Clinical and laboratory evaluation

Laboratory assessments included a complete blood count, serum C-reactive protein (CRP) using a Beckman Coulter AU5822/U.S analyzer (Beckman Coulter, Chaska, MN, USA), erythrocyte sedimentation rate (ESR), liver and renal function tests, 25-OH vitamin D (Abbott Alinity/U.S. analyzer) and urinary analysis.

25-OH vitamin D serum levels were classified as deficiency if under 20 ng/mL.

C-reactive protein levels were categorized as follows: values <5 mg/L were considered within the normal range, levels between 5 and <15 mg/L were classified as moderate elevation, and levels >15 mg/L were considered high.

The Disease Activity index for Psoriatic Arthritis is a validated tool specifically designed to assess disease activity in patients with psoriatic arthritis. DAPSA incorporates clinical parameters such as tender and swollen joint counts, patient global assessment, patient pain assessment, and C-reactive protein levels to provide a comprehensive evaluation of disease activity. The scoring system categorizes disease activity into different levels: ≤4 (remission), >4 – ≤14 (low disease activity), >14 – ≤28 (moderate disease activity), >28 (high disease activity) (13).

### 2.3 Imaging evaluation and technique

The analysis aimed to elucidate potential differences in clinical characteristics and outcomes between these two patient populations.

Ultrasound imaging was performed using a GE Logiq P9 sonograph (GE Healthcare, Chicago, IL, USA), equipped with a musculoskeletal probe (frequency range: 3–12 MHz) to facilitate detailed visualization of musculoskeletal structures. Color and Power Doppler imaging capabilities were utilized to assess vascular flow within the tissues, enabling the identification of blood flow characteristics and perfusion patterns.

This modality enables detailed visualization of superficial articular and periarticular structures, including synovium, entheses and tendons. Both grayscale (B-mode) and Power Doppler imaging were utilized to assess structural integrity and active inflammation, respectively.

Ultrasound synovitis was graded semi-quantitatively according to OMERACT criteria on a scale from Grade 0 (no synovitis) to Grade III (severe synovitis). These ordinal grades (US1 at baseline and US2 at 6 months) were also converted into numerical scores to facilitate quantitative analyses, including calculation of mean values and correlation tests with NVC and clinical parameters (14).

The ultrasonographic examinations were performed by D.A., an experienced physician in musculoskeletal ultrasonography.

For microvascular assessment, a DS Medica Videocap 3.0 (DS Medica, Milan, Italy), videocapillaroscope was used. It offers high-resolution imaging of superficial capillaries, with features such as magnification up to 200x, electronic image capture, and image analysis software for visual assessment of capillary density, morphology, and flow.

The examination was performed in the nailfold region, where capillaries run parallel to the skin surface, facilitating visualization.

The examination included digits 2–5 bilaterally, in accordance with standard capillaroscopic practice. Multiple fields were captured for each digit to allow adequate visualization of the nailfold microcirculation and representative assessment of capillary morphology and density.

Patients were acclimatized prior to the examination under standardized room temperature conditions, following The European Alliance of Associations for Rheumatology - EULAR Study Group recommendations for microcirculation assessment, in order to minimize temperature-related vascular variability.

The NVC examinations were performed by OGP. Image interpretation and analysis were independently carried out by MLG and OGP using the DS Medica image acquisition and analysis software integrated into the Videocap 3.0 system. Although interobserver reliability of NVC image evaluation was not specifically analyzed, standardized assessment procedures were applied to support consistency in image interpretation (predefined morphological parameters as stated below).

Parameters, including capillary density, length, width, architectural arrangement, tortuosity, presence of angiogenesis, microhemorrhages and visibility of the subpapillary venous plexus, were systematically evaluated. Capillary loop width exceeding 20 µm was classified as dilated, while widths greater than 50 µm were categorized as giant or megacapillaries. Capillary lengths exceeding 300 µm were categorized as elongated. Normal capillary density indicates ≥7 capillaries/mm, whereas values below this threshold denote low density. Avascular areas were identified when the intercapillary distance exceeded 500 µm. The presence of angiogenesis is identified as highly tortuous or “bushy/coiled” capillary configurations, indicative of neovascularization following capillary loss (15).

The videocapillaroscopic parameters were assessed using a combination of qualitative and semi-quantitative approaches based on standardized definitions used in nailfold videocapillaroscopy. Capillary density was measured by counting the number of capillaries per millimeter. Dilated and giant capillaries were identified using established diameter thresholds, while avascular areas were defined by a clear reduction or absence of capillaries within the examined field.

Dilated capillaries/megacapillaries and microhemorrhages were recorded qualitatively as present or absent (binary coding). Other capillaroscopic abnormalities were assessed using a semiquantitative scoring system based on their extent within the examined field: score 0 = absent, score 1 = involvement of <33% of capillaries (mild), score 2 = involvement of 33–66% of capillaries (moderate), and score 3 = involvement of >66% of capillaries (severe). When there was uncertainty in interpretation of any image, the findings were reviewed jointly by the observers (OGP and MLG) and a final classification was reached by consensus (16).

### 2.4 Statistical analysis

For these patients, data were collected on several variables, including sex, age, videocapillaroscopy measurements taken at baseline and after six months of treatment, ultrasound assessments conducted at baseline (US1) and after six months of treatment (US2), as well DAPSA scores, 25-OH Vitamin D levels, and C-reactive protein (CRP) levels.

The analysis of the possible correlations between these parameters was performed using Statistical Package for Social Sciences (SPSS), version 20 (IBM Corp., New York, NY, USA) and the following statistical tools: Pearson correlation coefficient, Student-t and Repeated measures. Chi-square tests (χ2 test) were used for categorical data. For the present study, the following p values were accepted: p < 0.05 significant in a confidence interval (CI) of 95%, p < 0.01 (CI of 99%), p < 0.001 highly significant (CI of 99.9%).

Associations between categorical variables, including the presence or absence of dilated capillaries, were evaluated using the Chi-square test.

Associations between categorical variables were evaluated using the Chi-square test. Differences in ordinal ultrasound synovitis grades (OMERACT 0–III) according to NVC abnormalities were assessed using the Mann–Whitney U test, as appropriate for comparisons between categorical NVC variables and ordinal, non-normally distributed data.

## 3 Results

A retrospective observational study was conducted on 75 patients—40 diagnosed with psoriatic arthritis with distal interphalangeal involvement (mean disease duration 3.23 ± 2.15 years) and 35 with psoriasis alone (mean disease duration 9.37 ± 5.48 years). Initially, 85 patients were considered, but 45 met exclusion criteria. The participants were divided into two groups: the psoriatic arthritis group (Group 1) and the control group (Group 2) – **Figure 1**.

In cohort 1, we evaluated a total of 40 patients diagnosed with psoriatic arthritis who initiated TNF inhibitors (Infliximab, Adalimumab or Etanercept) and were evaluated after 6 months by their physicians. The anti-TNF agents included infliximab (n = 9), adalimumab (n = 16) and etanercept (n = 15).

**Figure 2:**
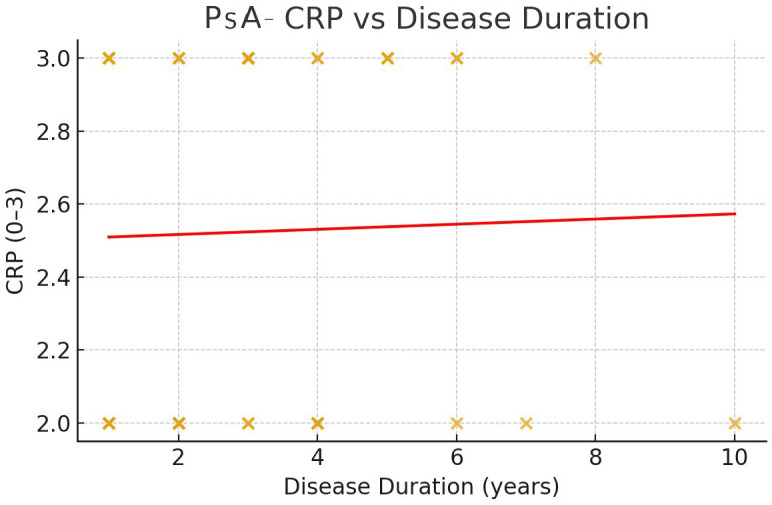
Relationship between disease duration and CRP levels in the psoriatic arthritis cohort.

The cohort consisted of 20 men (50%) and 20 women (50%). The mean age of the cohort was 45.5 years. Regarding disease duration, there was a tendency toward higher inflammatory scores and a greater number of NVC abnormalities with increasing disease duration – **Figure 2**.

Patients with low disease activity according to DAPSA showed better videocapillaroscopic values.

In terms of 25-OH Vitamin D levels, 17 patients (42.5%) had normal levels, whereas 23 patients (57.5%) had low levels (<20 ng/mL). The CRP results revealed that 19 patients (47.5%) had moderate levels, while 21 patients (52.5%) exhibited very high levels of CRP.

Tortuous capillaries and low capillary density correlated with high CRP values (p = 0.0031) and respectively p = 0.0030.

The presence of certain NVC abnormalities was significantly associated with higher disease activity (high DAPSA scores): tortuous capillaries (p = 0.0445), low capillary density (p = 0.00855) and dilated capillaries (p = 0.0463).

**Figure 3:**
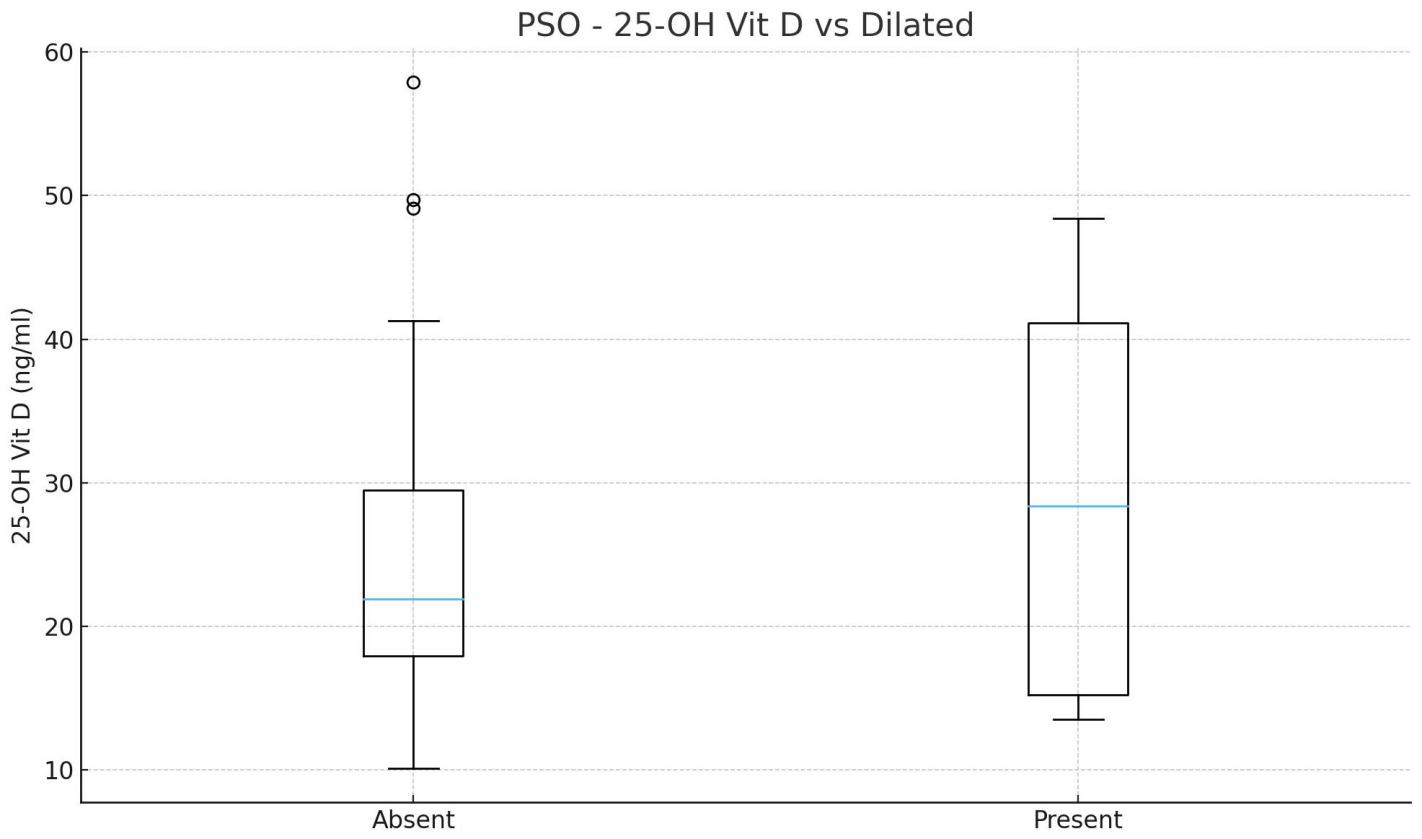
Boxplots comparing serum 25-OH vitamin D concentrations between patients with and without dilated capillaries.

Moreover, high CRP levels correlated with DAPSA scores (p = 0.0204) and showed a strong correlation with low 25-OH vitamin D levels. Patients with dilated capillaries showed significantly lower 25-OH vitamin D levels compared with those without dilated capillaries. Differences were assessed using the Mann–Whitney U test (*p* < 0.0001) – **Figure 3**. Additionally, vitamin D deficiency was associated with severe synovitis and elevated CRP.

No significant correlations were made between neoangiogenesis and CRP, 25-OH vitamin D levels. Other NVC features (crossed capillaries, megacapillaries, hemorrhages) did not show statistically significant correlations with synovitis, CRP, vitamin D or DAPSA in the PsA cohort.

Dilated capillaries positively correlated with ultrasound synovitis grade (p = 0.0314) and with high CRP levels (p = 0.0039) and inversely correlated with 25-OH vitamin D levels (p = 0.0079).

Specific NVC features (neoangiogenesis, hemorrhages, dilated capillaries) were more common in severe synovitis.

Patients with PSA demonstrated higher tortuous capillary scores, with a significant reduction at 6 months (p = 0.0391). The capillaroscopic findings in psoriatic arthritis cohort at baseline vs at 6 months were presented in **Table 1**.

**Table 1:** Psoriatic arthritis cohort at baseline vs at 6 months.

Variable	PsA baseline	PsA 6 months	Difference (↓)
Disorganised	15 (37.5%)	8 (20.0%)	-17.5% (↓↓)
Venous Plexus	4 (10.0%)	4 (10.0%)	0.0%
Tortuous	14 (35.0%)	9 (22.5%)	-12.5% (↓)
Crossed	9 (22.5%)	4 (10.0%)	-12.5% (↓)
Dilated	13 (32.5%)	8 (20.0%)	-12.5% (↓)
Megacapillaries	3 (7.5%)	1 (2.5%)	-5.0% (↓)
Neoangiogenesis	5 (12.5%)	1 (2.5%)	-10.0% (↓)
Hemorrhages	2 (5.0%)	0 (0.0%)	-5.0% (↓)
Low density	18 (45.0%)	13 (32.5%)	-12.5% (↓)

↓ = decreased. ↓↓ = significantly decreased.

DIP enthesitis was associated with greater capillary architectural disorganization, observed both at baseline (p = 0.0005) and at 6 months (p = 0.0483).

Patients with low 25-OH Vitamin D levels (who had received vitamin D supplementation) showed a more favorable improvement in NVC parameters after six months of biological treatment and Vitamin D supplementation, especially regarding tortuous capillaries. Higher Vitamin D levels correlate with better NVC parameters.

Biologic therapy produced selective but meaningful effects on microvascular changes over the 6-month follow-up. Tortuous capillaries were the only feature to significantly improve (p = 0.0364), indicating early microvascular normalization following treatment initiation. In contrast, other capillaroscopic parameters—including crossed capillaries (p = 0.7505), angiogenesis (p = 0.2162), capillary architecture (p = 0.9074), and dilated capillaries (p = 1.0000)—did not show statistically significant changes. Capillary density remained unchanged. Correlation analyses highlighted several clinically relevant associations: at baseline, capillary density correlated inversely with DAPSA disease activity (p = 0.0239). At 6 months, angiogenesis showed a strong positive correlation with CRP (p = 0.0156), capillary density inversely correlated with DAPSA (p = 0.0239). Capillary disorganisation demonstrated a significant positive association with DAPSA scores (p = 0.0307).

No significant differences were observed among the three anti-TNF agents. The evaluated parameters across agents were: capillary density, length, width, architectural arrangement, tortuosity, presence of angiogenesis, microhemorrhages.

In Cohort 2 (control group), comprising of patients with psoriasis vulgaris, we present findings related to videocapillaroscopy parameters, measured at baseline and after 6 months of treatment. Capillaroscopic parameters are presented in **Table 2**.

**Table 2:** Comparison of psoriasis vulgaris cohort at baseline and at six months.

Variable	PSo baseline	PSo after 6 months	Difference
Disorganised	3 (8.5%)	7 (20.0%)	+ 11.4% (↑)
Venous Plexus	4 (11.4%)	4 (11.4%)	0%
Tortuous	18 (51.4%)	12 (34.29%)	-17.14% (↓↓)
Crossed	4 (11.4%)	5 (14.2%)	- 2.86% (↓)
Dilated	6 (17.1%)	6 (17.1%)	0%
Megacapillaries	0 (0.0%)	0 (0%)	0%
Neoangiogenesis	1 (2.8%)	1 (2.8%)	0%
Hemorrhages	2 (5.7%)	2 (5.7%)	0%
Low density	8 (22.8%)	7 (20.0%)	-2.8% (↓)

↑ = increased.

The study included 35 patients, of which 22 were males (62.9%) and 13 were females (37.1%). The mean age of the cohort was 49.74 years.

Longer disease duration was moderately associated with poorer NVC results (capillary density, capillary dilatation, capillary disorganization) and lower Vitamin D.

Among those with low vitamin D, 8 patients (44.4%) showed no change in their capillaroscopic pattern, while 10 (55.6%) showed improvement; of these, 3 (16.7%) achieved complete normalization and 7 (38.9%) showed partial improvement after six months.

In contrast, among the 17 patients with normal vitamin D levels, 4 patients (23.52%) showed improvement in their capillaroscopic pattern and 13 patients (76.47%) had no change.

A greater proportion of patients with low 25-OH-vitamin D levels showed reduced capillary density compared with those who had normal vitamin D levels at baseline (p=0.043).

Correction of 25-OH-Vitamin D deficiency was associated with improved capillary architecture (p=0.049).

To assess the role of systemic inflammation on nailfold microvascular changes, we compared baseline and 6-month videocapillaroscopic findings between patients with psoriatic arthritis (Group 1) and those with psoriasis vulgaris without joint involvement (Group 2). At baseline, patients with psoriasis vulgaris had better capillaroscopic values compared to those with psoriatic arthritis, suggesting more prominent microvascular alterations in the psoriatic arthritis group. After 6 months of treatment, patients in the psoriatic arthritis group showed a greater relative improvement in microvascular architecture.

When stratifying by vitamin D status, differences in microvascular opsotcomes became more pronounced. In Group 1, patients with low vitamin D who received supplementation showed significantly videocapillaroscopic improvements compared to those with normal levels.

No statistically significant differences in NVC evolution were observed when stratifying by sex or age in either group.

Comparison of psoriasis vulgaris cohort at baseline and at six months as well as the comparison between psoriatic arthritis and psoriasis vulgaris cohorts was presented in **Tables 1**, **2** respectively.

At baseline, patients in Cohort 1 (PsA) demonstrated more prominent microvascular abnormalities on videocapillaroscopy compared with Cohort 2 (PsO), characterized by higher frequencies of tortuous capillaries, greater capillary dilation, and reduced capillary density.

In PsA, the presence of tortuous capillaries was further associated with distal interphalangeal (DIP) enthesitis and elevated C-reactive protein levels, indicating a closer relationship between microvascular dysfunction and systemic inflammation. In contrast, patients in Cohort 2 exhibited slightly better baseline videocapillaroscopy scores, with fewer structural microvascular alterations. Overall, patients with PsA showed significantly more pronounced baseline microvascular changes than the PsO group. After six months, patients with PsA demonstrated selective improvement—primarily in tortuous capillaries—consistent with the anti-inflammatory effects of biologic therapy and the reduction of disease activity. Patients with PsO exhibited a more uniform improvement in NVC parameters, a response pattern more strongly influenced by baseline vitamin D status. Notably, in PsA, the combination of biologic therapy and vitamin D supplementation appeared to enhance microvascular recovery, whereas in PsO, baseline vitamin D concentration emerged as the predominant determinant of NVC improvement. Comparison between psoriatic arthritis and psoriasis vulgaris cohorts was presented in **Table 3**.

**Table 3:** Comparison between psoriatic arthritis and psoriasis vulgaris cohorts.

Feature	Cohort 1 (PsA)	Cohort 2 (PsO)	Key difference
Number	40	35	PsA vs PsO
Sex	50% M/F	62.9% M, 37.1% F	Slight male predominance in PsO
Disease duration	3.23 ± 2.15	9.37 ± 5.48	PsO slightly older
Disease activity	DAPSA (low/moderate/high)	Not applicable	Assessed only in PsA
25-OH Vitamin D effect	Correcting the 25 OH deficiency + biologics improved NVC values	Correcting the 25-OH deficiency slightly improved NVC values	PsA improvements required biological treatment; PsO depended on baseline 25-OH Vitamin D levels
Ultrasound	Present (synovitis/enthesitis grades)	Not assessed	Only PsA had joint correlation
Biologic therapy influence	TNF effects observed	Not applicable	PsA response therapy-specific

## 4 Discussions

The findings of our study provide important insights into the microvascular changes observed in patients with distal interphalangeal predominant psoriatic arthritis (PsA) undergoing treatment with tumor necrosis factor (TNF) inhibitors, as assessed through nailfold videocapillaroscopy (NVC). Our investigation highlights both the potential benefits of TNF inhibitors on microvascular health and the implications for disease monitoring in this specific patient population.

The demographic and clinical characteristics of the cohort in this study revealed several noteworthy findings regarding patients with Psoriatic Arthritis (PsA) and those with psoriasis.

The sex distribution among the patients with PsA was balanced, with an average age of approximately 45.5 years, as well as in the psoriasis group, averaging 49.7 years. In terms of treatment efficacy, significant improvements were observed in the ultrasonographic findings of patients with PsA after six months of TNF inhibitor therapy.

Patients with low disease activity, as measured by DAPSA, demonstrated more favorable videocapillaroscopic findings compared to those with higher disease activity. This suggests a potential correlation between systemic inflammation control and improved microvascular health, as observed through videocapillaroscopy.

An interesting observation concerning vitamin D levels and treatment outcomes was made. Patients with low 25-OH Vitamin D levels who received Vitamin D supplementation demonstrated a more favorable outcome in terms of NVC scores. A significant correlation (p=0.037) was identified, suggesting that correction of Vitamin D deficiency may be linked to better improvements in NVC. On the other hand, patients with normal Vitamin D levels exhibited a higher rate of stagnation in NVC results.

Vitamin D exerts multiple immunomodulatory functions, inhibiting pro-inflammatory cytokine release and osteoclastogenesis in PsA and psoriasis, suggesting it could have a natural biological impact on disease activity and microvascular health. These effects may extend to the enthesis organ, particularly at the distal interphalangeal level, which is characteristically affected in PsA (14).

The synergistic effect of biologic therapy and vitamin D supplementation in PsA raises the possibility that optimizing vitamin D levels may enhance the vascular and clinical benefits of biologic agents.

While direct studies of vitamin D status in relation to DIP enthesitis are lacking, the immunoregulatory role of vitamin D and its known vascular and bone tissue effects suggest potential mechanistic links. Lower vitamin D levels may impair endothelial resilience and immune regulation within the enthesis, thereby influencing the development of structural and vascular changes detectable by videocapillaroscopy (17).

Higher CRP values correlated significantly with several individual NVC abnormalities—including tortuous capillaries, low capillary density, and dilated capillaries. CRP reflected inflammatory microvascular involvement at baseline but it may not be a reliable marker for predicting microvascular improvement. Another review observed that CRP measurement is often inconsistently used in PsA trials, limiting its utility in tracking disease progression and microvascular improvement (18). Our study suggests that overall burden of videocapillaroscopic abnormalities together with systemic inflammation (CRP) and Vitamin D deficiency are important predictors of severe synovitis.

In patients with PsA, baseline NVC parameters correlate with ultrasound findings, particularly indicating that severe capillary alterations predict a more severe synovitis.

Among the evaluated NVC abnormalities, dilated capillaries demonstrated a significant positive association with ultrasound synovitis grade (p = 0.0314), indicating that patients with more pronounced capillary dilation tended to present more severe DIP joint inflammation. Furthermore, neoangiogenesis, hemorrhages, and dilated capillaries were observed more frequently in patients with severe synovitis, supporting a relationship between microvascular alterations and inflammatory joint severity. In contrast, crossed capillaries, megacapillaries and hemorrhages did not demonstrate statistically significant associations with synovitis grade, suggesting that these capillaroscopic abnormalities may not consistently reflect the degree of inflammatory joint activity in this PsA cohort.

Previous studies have identified significant differences in capillary density, morphology, and perfusion in patients with PsA compared to healthy controls (19).

In our study, nailfold videocapillaroscopic parameters correlate with the severity of distal interphalangeal joint involvement. The findings suggest that microvascular changes may serve as a biomarker for disease activity and severity, particularly in patients with predominant distal interphalangeal involvement.

Fukasawa et al. observed that the degree of NVC abnormalities correlated with the severity of PsA and serum cytokine levels, including TNF, IL-17A, and IL-23. These findings suggest that NVC abnormalities can serve as predictive markers for the development and severity of PsA in psoriasis patients (20).

Anghel et al. demonstrated notable alterations in nailfold capillaroscopic patterns among patients with PsA, including reduced capillary density, increased tortuosity, architectural disorganization, with the occasional presence of microhemorrhages and dilated capillaries. After 12 months of treatment, there was a marked improvement in capillary parameters, including increased density, reduction in dilated and giant capillaries, normalization of capillary length, and decreased signs of angiogenesis. These features underscore the role of microvascular dysfunction in the disease pathogenesis (21).

A recent study showed that 60.1% of patients with PsA with nail abnormalities had extensor tendon enthesopathy detected with the help of ultrasonography compared to those with no apparent nail lesions (22%) (22, 23). This information underscores the necessity for routine imaging in at-risk populations, as early detection facilitates a more proactive approach to management and may ultimately reduce healthcare costs associated with more advanced disease. Thus, integrating imaging techniques such as nailfold capillaroscopy and ultrasound in the management of psoriatic disease is essential for optimizing patient care and improving prognostic outcomes.

Our results indicate that treatment with TNF inhibitors is associated with a notable improvement in NVC parameters, including increased capillary density and normalization of capillary morphology. These findings are consistent with existing literature suggesting that TNF inhibitors can positively influence vascular function and inflammation in autoimmune diseases. The observed improvements in capillary parameters may reflect a reduction in the underlying inflammatory processes associated with PsA.

Nailfold videocapillaroscopy has emerged as a valuable, non-invasive imaging technique for assessing microvascular involvement in systemic autoimmune diseases, with growing evidence supporting its diagnostic and monitoring utility in several diseases, including immune-mediated rheumatic diseases and even interstitial lung disease and diabetes mellitus (24, 25).

Overall, microvascular alterations were more marked in patients with PsA than in those with PsO. Patients with PsA primarily showed improvement in tortuous capillaries, suggesting benefits from biologic therapy and reduced disease activity. Notably, in PsA, the combination of biologic therapy and vitamin D supplementation appeared to further support microvascular recovery, whereas in PsO, baseline vitamin D emerged as the main driver of NVC improvement.

Despite the promising findings, our study is not without limitations. The sample size, while adequate for preliminary analysis, could restrict the applicability of our results. Longitudinal studies with larger cohorts are necessary to further elucidate the relationship between TNF inhibitor therapy and microvascular alterations in PsA.

The observed improvements in NVC findings in the control group should be interpreted with caution, as factors other than vitamin D supplementation may have contributed to these changes. In addition, concomitant therapies, including anti-TNF treatment in the patient group, may have exerted independent effects on microvascular alterations and represent potential confounding factors. Future studies with larger sample sizes and appropriate multivariate analyses are needed to better elucidate the association between vitamin D levels and disease activity and to distinguish these effects from those of therapeutic interventions.

## 5 Conclusion

Our findings demonstrate a clear association between nailfold videocapillaroscopic abnormalities and the extent of distal interphalangeal joint involvement. In PsA, improvements in capillary parameters likely reflect the effects of biologic therapy combined with vitamin D supplementation, while in PsO, correcting vitamin D levels appears to be the main factor driving vascular improvement. These results support the potential utility of microvascular alterations as surrogate markers for disease activity and severity, particularly in patients with dominant distal interphalangeal joint pathology.

Nailfold videocapillaroscopy is a valuable tool for assessing microvascular changes in patients with distal interphalangeal predominant psoriatic arthritis treated with TNF inhibitors. The observed improvements in capillary parameters highlight the potential of TNF inhibitors to positively impact microvascular health, suggesting that NVC may serve as a useful complementary tool in the management of PsA.

Moreover, these findings support integrating vitamin D assessment—and potentially supplementation—into the management strategy for patients with psoriatic disease. Further research is warranted to solidify the role of NVC in clinical practice and to enhance our understanding of how vascular changes and disease progression in psoriatic arthritis are linked.

## Data Availability

The raw data supporting the conclusions of this article will be made available by the authors, without undue reservation.
