# Supplementary figures and images for: Correlations between serum 25-hydroxyvitamin D levels and nailfold microvascular changes in psoriatic arthritis patients with distal interphalangeal arthritis treated with TNF inhibitors
Source: Front Immunol. 2026 Jul 6;17:1847741. doi: 10.3389/fimmu.2026.1847741 (PMC13381474; doi:10.3389/fimmu.2026.1847741)

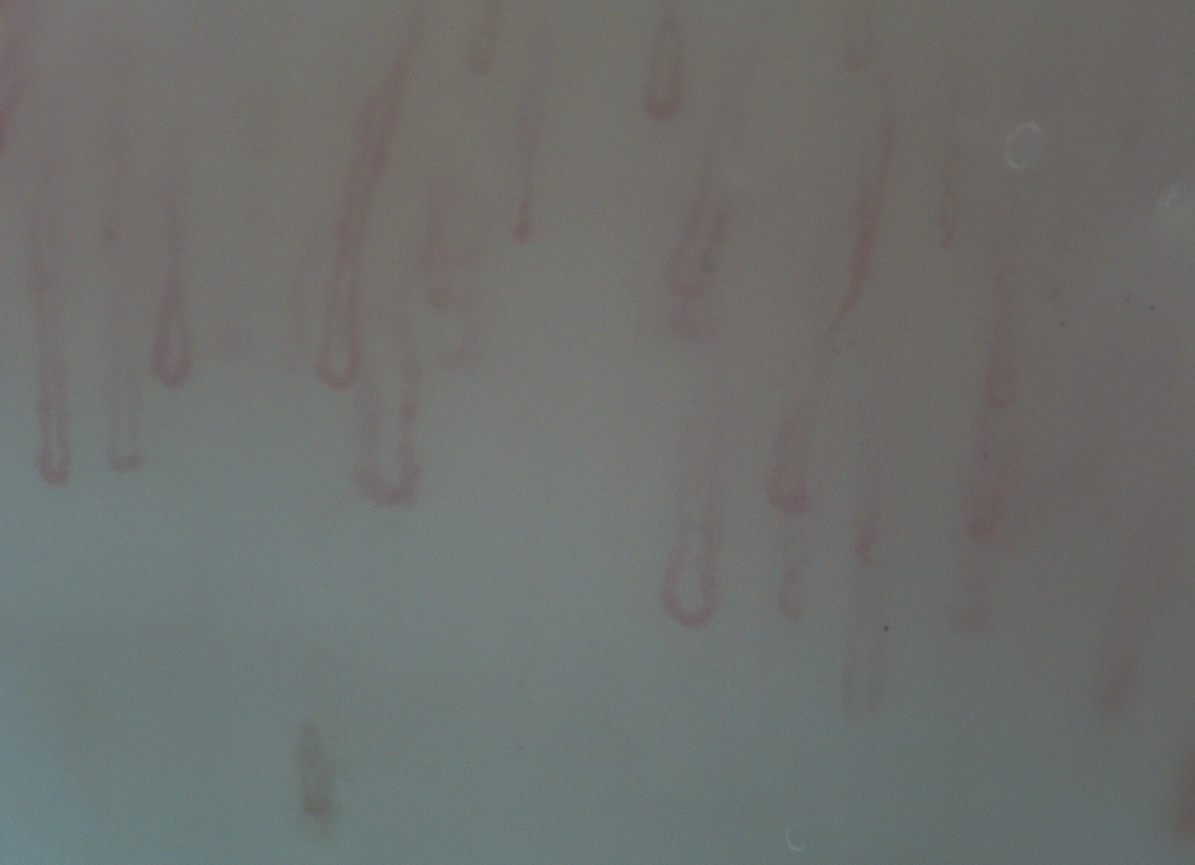

Supplement: Supplementary file 1 [file Image1.png]

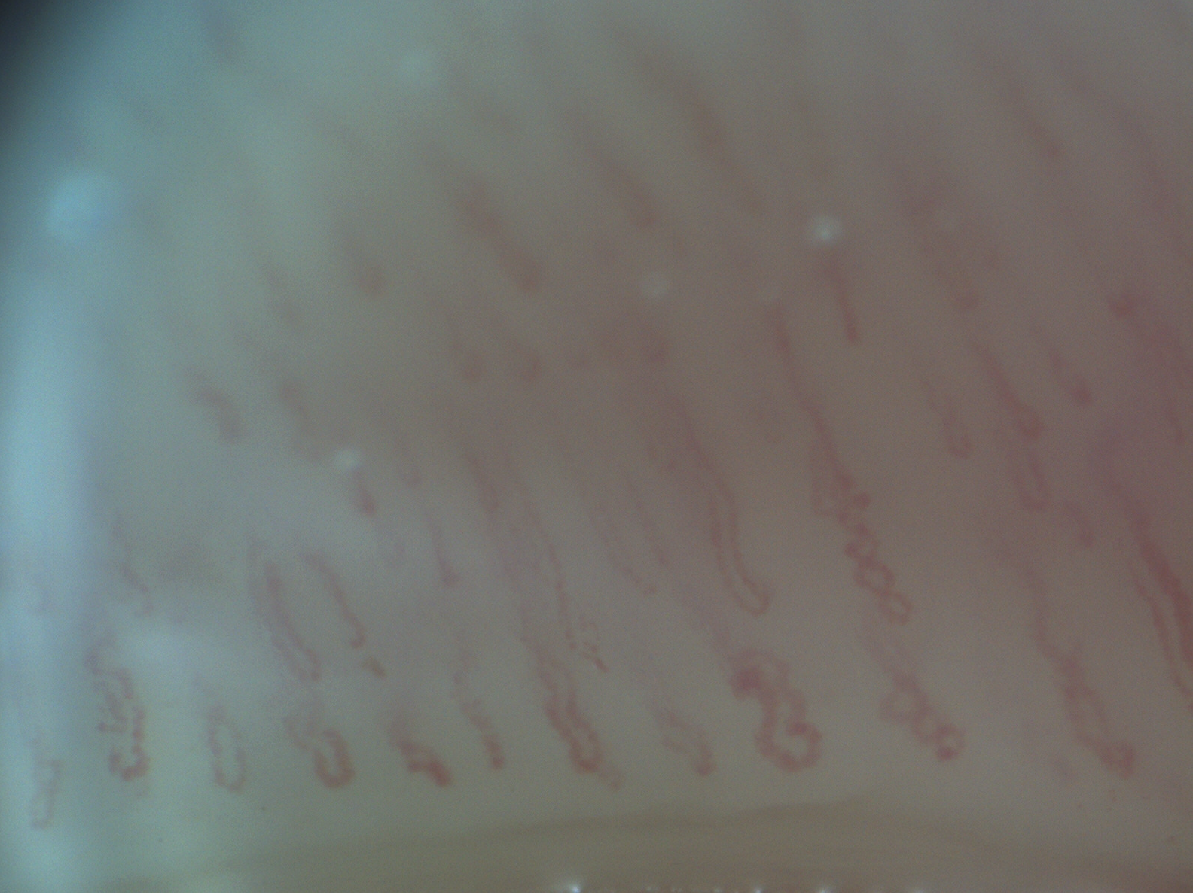

Supplement: Supplementary file 2 [file Image2.png]

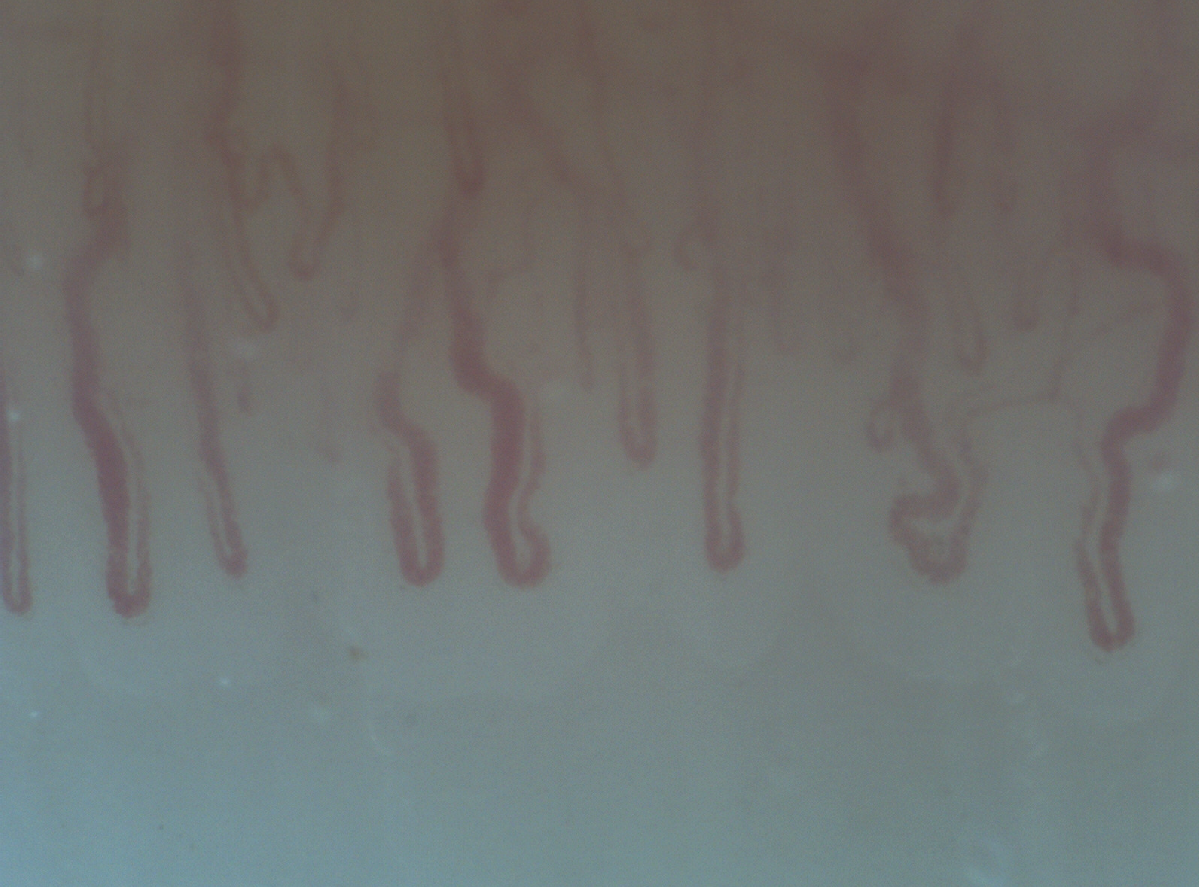

Supplement: Supplementary file 3 [file Image3.png]

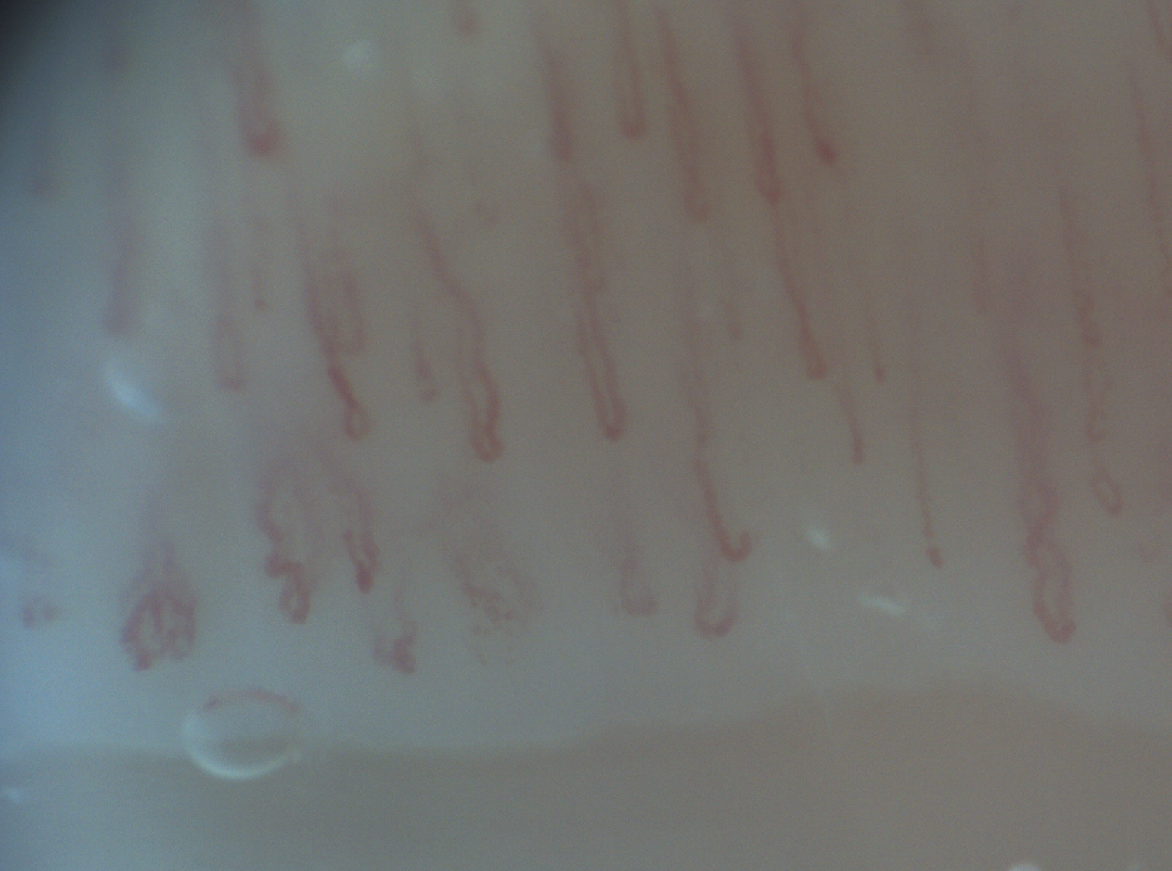

Supplement: Supplementary file 4 [file Image4.png]

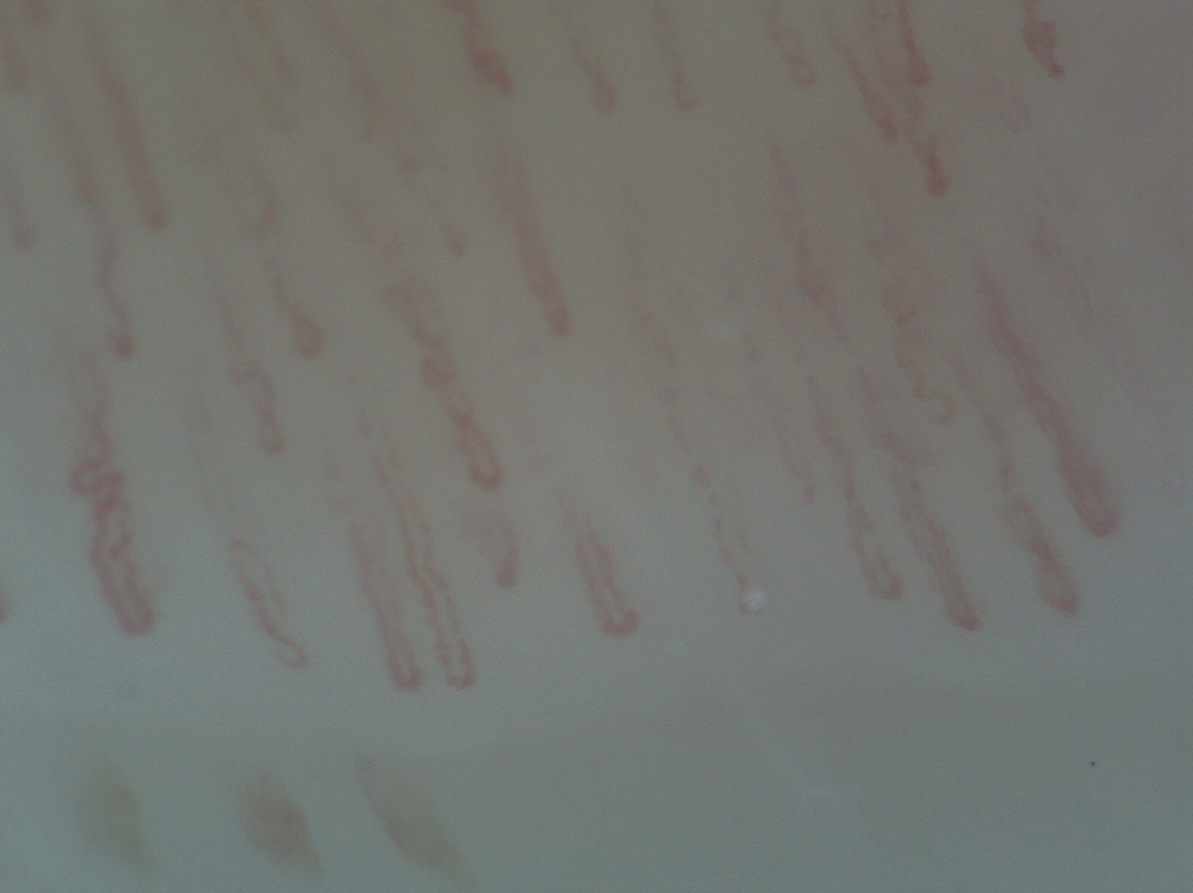

Supplement: Supplementary file 5 [file Image5.png]
